# Novel Biomarkers and Their Role in the Diagnosis and Prognosis of Acute Coronary Syndrome

**DOI:** 10.3390/life13101992

**Published:** 2023-09-29

**Authors:** Maria Katsioupa, Islam Kourampi, Evangelos Oikonomou, Vasiliki Tsigkou, Panagiotis Theofilis, Georgios Charalambous, George Marinos, Ioannis Gialamas, Konstantinos Zisimos, Artemis Anastasiou, Efstratios Katsianos, Konstantinos Kalogeras, Ourania Katsarou, Manolis Vavuranakis, Gerasimos Siasos, Dimitris Tousoulis

**Affiliations:** 1 3rd Department of Cardiology, Thoracic Diseases General Hospital “Sotiria”, National and Kapodistrian University of Athens, 11527 Athens, Greece; mkatsioupa@gmail.com (M.K.); kourampiislam@gmail.com (I.K.); boikono@gmail.com (E.O.); bikytsigkoy@yahoo.gr (V.T.); jyialamas@gmail.com (I.G.); zisimoskostas@gmail.com (K.Z.); artemisan93@gmail.com (A.A.); stratiskatsianos@yahoo.com (E.K.); kalogerask@yahoo.gr (K.K.); raniakatsarou@yahoo.gr (O.K.); vavouran@otenet.gr (M.V.); 2 1st Department of Cardiology, “Hippokration” General Hospital, National and Kapodistrian University of Athens, 11527 Athens, Greece; panos.theofilis@hotmail.com (P.T.); drtousoulis@hotmail.com (D.T.); 3 Department of Emergency Medicine, “Hippokration” General Hospital, National and Kapodistrian University of Athens, 11527 Athens, Greece; drcharalambous@yahoo.gr; 4 Department of Hygiene, Epidemiology and Medical Statistics, Medical School, National and Kapodistrian University of Athens, 11527 Athens, Greece; marinosgiorgos@hotmail.com

**Keywords:** biomarker, acute coronary syndrome, coronary artery disease, myocardial injury, neurohormonal activation, inflammation, thrombosis

## Abstract

The burden of cardiovascular diseases and the critical role of acute coronary syndrome (ACS) in their progression underscore the need for effective diagnostic and prognostic tools. Biomarkers have emerged as crucial instruments for ACS diagnosis, risk stratification, and prognosis assessment. Among these, high-sensitivity troponin (hs-cTn) has revolutionized ACS diagnosis due to its superior sensitivity and negative predictive value. However, challenges regarding specificity, standardization, and interpretation persist. Beyond troponins, various biomarkers reflecting myocardial injury, neurohormonal activation, inflammation, thrombosis, and other pathways are being explored to refine ACS management. This review article comprehensively explores the landscape of clinically used biomarkers intricately involved in the pathophysiology, diagnosis, and prognosis of ACS (i.e., troponins, creatine kinase MB (CK-MB), B-type natriuretic peptides (BNP), copeptin, C-reactive protein (CRP), interleukin-6 (IL-6), d-dimers, fibrinogen), especially focusing on the prognostic role of natriuretic peptides and of inflammatory indices. Research data on novel biomarkers (i.e., endocan, galectin, soluble suppression of tumorigenicity (sST2), microRNAs (miRNAs), soluble oxidized low-density lipoprotein receptor-1 (sLOX-1), F2 isoprostanes, and growth differentiation factor 15 (GDF-15)) are further analyzed, aiming to shed light on the multiplicity of pathophysiologic mechanisms implicated in the evolution of ACS. By elucidating the complex interplay of these biomarkers in ACS pathophysiology, diagnosis, and outcomes, this review aims to enhance our understanding of the evolving trajectory and advancements in ACS management. However, further research is necessary to establish the clinical utility and integration of these biomarkers into routine practice to improve patient outcomes.

## 1. Introduction

Cardiovascular diseases continue to pose a significant burden on overall mortality worldwide [1,2] and acute coronary syndrome (ACS) is often the initial presentation. ACS, encompassing a spectrum of conditions from unstable angina (UA) to ST-segment elevation myocardial infarction (STEMI), presents a critical challenge in contemporary cardiology. ACS results from the disruption of atherosclerotic plaques within the coronary arteries, leading to myocardial ischemia, necrosis, and subsequent clinical presentations [3]. Early diagnosis and risk stratification are therefore critical for guiding timely interventions and improving patient outcomes [4].

**Figure 1:**
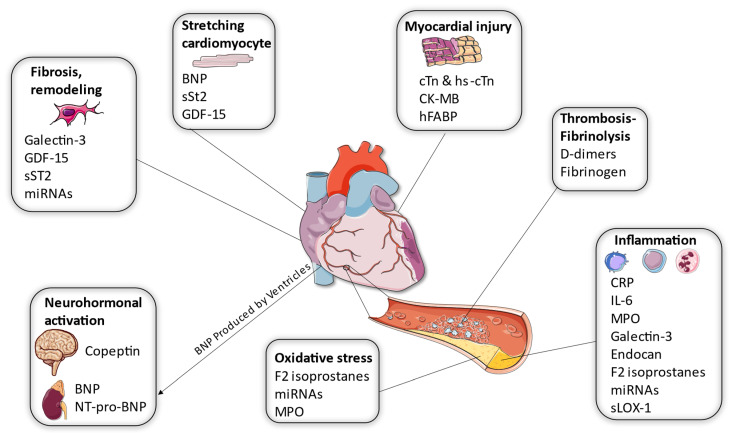
Different roles of biomarkers in the pathophysiology of Myocardial Infarction. The biomarkers for MI can be categorized based on their mechanism of action. Some are secreted by the cardiomyocytes, while others are correlated with inflammation, clot formation, and neurohormonal pathways. It is interesting to note that some biomarkers (galectin-3, miRNAs, BNP, sST2, and GDF-15) are included in more than one category, proving the multiplicity of their pathophysiology (BNP: B type natriuretic peptide; CK-MB: Creatine Kinase- MB; CRP: reactive protein; cTn: Cardiac Troponin; GDF-15: Growth Differentiation Factor-15; hFABP: Heart- Type Fatty Acid Binding Protein; MI: Myocardial Infarction; miRNAs: micro-RNAs; MPO: Myeloperoxidase; NT-proBNP: N-terminal portion of the pro-BNP peptide; sLOX-1: Soluble oxidized low-density lipoprotein receptor-1; sST2: Soluble Suppression of Tumorigenicity) Parts of the figure were drawn by using pictures from Servier Medical Art. Servier Medical Art by Servier is licensed under a Creative Commons Attribution 3.0 Unported License (https://creativecommons.org/licenses/by/3.0/) (accessed on 16 September 2023).

Biomarkers have emerged as essential endpoints for the diagnosis, risk stratification, and prognosis assessment of ACS. Among these biomarkers, troponin, particularly high-sensitivity troponin (hs-cTn), has revolutionized ACS diagnosis with its superior sensitivity and negative predictive value [5]. However, challenges such as specificity, standardization, and interpretation persist. Beyond troponins, a constellation of biomarkers reflecting myocardial injury, neurohormonal activation, inflammation, thrombosis, and other pathways are being explored to refine ACS management (Figure 1).

Therefore, in this review article, we focus on elucidating the elaborate landscape of biomarkers intricately involved in the pathophysiology, diagnostic process, and prognostic assessment of ACS, aiming to enhance our comprehension of the advancement of ACS as well as the clinical ramifications stemming from these biomarkers.

## 2. Biomarkers of Myocardial Injury

### 2.1. Troponin and High-Sensitivity Troponin

The troponin contractile apparatus consists of three isoforms (C, I, & T), which interact with tropomyosin, actomyosin, and Ca^2+^, regulate muscle contraction, and release from the necrotic myocardium (Table 1). Troponin C is not utilized in clinical practice as a biomarker since there is significant homology between the skeletal and cardiac isoforms, reducing cardiac specificity [6]. Since 2000, Troponin I and T has been a criterion ‘sine qua non’ for myocardial infarction (MI) diagnosis along with clinical and electrocardiography (ECG) conditions. Early conventional cTn assays, while specific, had a low sensitivity; analytical methods however have since evolved, with the hs-cTn assays (5th generation) prevailing as they offer higher diagnostic accuracy, an earlier detection of MI (within one hour), and a higher Negative Predictive Value (NPV) in order to rule out a MI (99.5–99.8%) [7,8]. These benefits in sensitivity and NPV come at the cost of specificity and Positive Predictive Value (PPV) compared to the conventional cTn (ruling in a MI), leading to more extensive testing [9,10]. Due to the high sensitivity, the ESC 2020 guidelines recommend the rapid ‘rule-out’ and ‘rule-in’ algorithms, especially the 0h/1h (alternatively 0 h/2 h) of serial drawing samples with a minimal sensitivity of 99% and a minimal PPV of 70%. In fact, Non-ST-Elevation Myocardial Infarction (NSTEMI) can be ruled out at baseline for a very low hs-cTn and/or lack of elevation within the first hour [7]. hs-cTn is also a quantitative biomarker in the diagnosis of MI as 5x Upper Reference Limit (URL) increases have a high PPV (>90%) for type 1 MI and this limit can differentiate from type 2 MI; at the same time, the increase in diagnosed MIs has led to a subtle reduction in the incidence of UA [7,11].

However, in a study there was a significant discordance in the results of 3 different hs-cTn assays, showcasing a lack of standardization between different manufacturers. This inconsistency was especially pronounced in troponin levels below the limit of detection (LOD) and between the LOD and the 99th percentile, while the proportion of samples above the 99th percentile did not fluctuate significantly between the clusters. As a result, the patients with rule-out and observe recommendations significantly differed between groups, while there was no significant difference in the rule-in candidates across different assays and research teams. This discrepancy can be attributed to differences in sensitivity and LOD or differences in the reference biobank used by each assay to determine the 99th percentile [8]. Standardization and harmonization issues between hs-cTn I assays are especially pronounced compared to hs-cTn T as Troponin I is offered by a variety of manufacturers; results between companies are therefore not interchangeable [6]. Concerning the differences between the isoforms T and I, cTn- I has a higher diagnostic accuracy in early presenters, while cTn-T prevails later [12,13]. Finally, cTn-T is superior in the prognosis of all-cause mortality, but its clearance is more heavily impacted in renal failure [13,14].

### 2.2. Creatine Kinase MB (CK-MB)

Creatine Kinase MB (CK-MB) has constituted the gold standard for MI diagnosis in the past [15] although the current European Society of Cardiology (ESC) guidelines do not recommend its routine measurement for diagnostic purposes [7]. Creatine Kinase metabolizes Creatine and Adenosine Triphosphate (ATP) to Creatine Phosphate and Adenosine Diphosphate (ADP) in muscle cells, while the CK-MB isoenzyme constitutes 20% of the overall myocardium CK pool. CK-MB starts rising 4–6 h after an MI, attains its apex at 24 h, and declines in 48–72 h, providing the possibility of early detection of a re-infarction [7,16]. During the first 6 h after a MI, CK-MB has a NPV of 97% and a 91% sensitivity. As CK-MB is released from skeletal muscle as well, it is important to avoid false positives in diagnosis by paying attention to the regularity and amount of secretion; a CK-MB:CK ratio > 2.5% increases the likelihood of cardiac sources [16]. Apart from its diagnostic properties, CK-MB adds prognostic characteristics as well, since peak CK-MB strongly correlates with the width of the lesion and wall movement anomalies in smaller non-transmural [17] and larger infarcts [18] after reperfusion. Additionally, in a study of participants 4 months after a STEMI, peak CK-MB was considered as a very strong predictor of the end systolic volume index assisting in prognosis of Left Ventricle (LV) remodeling [19]. Other useful associations consist of peak CK-MB levels correlating with 3-year mortality in NSTEMI after percutaneous coronary intervention (PCI) [20] and with the rare incidence of Heart Failure (HF) after a STEMI [21]. Finally, among MI patients undergoing coronary angiography, those with a preprocedural log (CK-MB) > 4.7 have an amplified incidence of contrast-induced acute kidney injury (CI-AKI) independently of other risk factors [22].

## 3. Biomarkers of Neurohormonal Activation

### 3.1. B Type Natriuretic Peptide (BNP)

BNP, a hormone generated due to myocardial dysfunction, is produced predominantly by the ventricles. Its functions encompass the hindrance of the renin-angiotensin-aldosterone system (RAAS), promotion of renal sodium excretion, and reduction in vascular resistance [23].

The concentration of BNP increases significantly within the initial 24 h following an MI in patients with STEMI and subsequently reaches a relatively stable level. There might be a second peak in BNP levels around 5 days later, possibly indicating the ongoing remodeling process [24].

Extensive research has been conducted on BNP, revealing its significance as a prognostic indicator following an MI [24,25,26]. BNP has a relatively short half-life, but it is secreted alongside the N-terminal section of the pro-BNP peptide (NT-proBNP), a fragment with a longer half-life in plasma and therefore more conveniently measured [27].

In a case-control study involving patients with non-ST-elevation ACS, it was discovered that NT-proBNP concentration was elevated in subjects who experienced mortality compared to those who survived [28]. NT-proBNP elevation occurs in the initial stages of ACS, particularly when angina lasts for less than 4 h [29]. It also exhibits a close association with the extent of myocardial ischemia and predicts both short- and long-term mortality among ACS cases [30]. In another study, NT-proBNP served as a marker strongly linked to all-cause mortality [31].

The TACTICS-TIMI 18 study [32] involved the randomization of 1676 patients with non-ST-elevation ACS into conservative and early invasive therapy groups. The study measured patients’ BNP levels within 24 h and compared the results. The findings revealed that patients with BNP levels below the cut-off of 80 pg/mL had a six-month mortality rate of 1.4%, whereas those with BNP levels above the cut-off had a mortality rate of 8.4%. Additionally, the risk of mortality or congestive heart failure was 3.6% for patients below the cut-off, compared to 16.3% for those above the cut-off. However, similar to another study [33], the TACTICS-TIMI 18 study did not determine which patients would have better outcomes from early invasive revascularization based on BNP levels.

### 3.2. Copeptin

Copeptin is a portion of the vasopressin precursor hormone (CT-proAVP) secreted alongside vasopressin after the precursor is cleaved. Measuring copeptin appears to be a clinically valuable approach for assessing plasma concentrations of vasopressin, which cannot be directly measured due to demanding pre-analytical techniques [34,35]. Studies have indicated that it could serve as a prognostic factor for STEMI [36]. In recent findings, copeptin has emerged as both an independent surrogate of total mortality and a marker of overall vulnerability, affected by the infliction of HF, type 2 diabetes, female sex, and prior MI [37]. In a prospective trial of patients with acute myocardial infarction (AMI), it was demonstrated that incorporating copeptin alongside cTn I enabled a secure exclusion of AMI with a NPV exceeding 99% in patients presenting early with suspected ACS. Moreover, the study indicated that the presence of both aberrant copeptin and cTn I levels was an independent indicator of mortality within 6 months [38]. In summary, recent evidence indicates that copeptin may offer supplementary benefits to cTn in promptly ruling out patients with suspected ACS [39].

## 4. Inflammatory Biomarkers

### 4.1. C Reactive Protein (CRP)

Atherothrombosis, a major contributor to ACS [40], mainly results from inflammatory processes [41]. CRP has been extensively researched as an indicator of acute-phase inflammation and could possibly assess increased cardiovascular risk in individuals with pre-existing atherosclerosis. According to the literature, plasma levels of CRP can estimate the likelihood of future MI and stroke [42]. Importantly, statins have the potential to decrease CRP levels beyond their cholesterol-lowering effects [43], making CRP a valuable test to reassess individuals categorized as having an intermediate risk for future cardiovascular events.

Moreover, patients diagnosed with UA and CRP levels greater than 3 mg/L upon discharge face an increased risk of readmission within one year for reoccurring cardiovascular instability or MI [44]. Likewise, in a prospective study of individuals who went through early invasive therapy for NSTEMI, elevated CRP levels exceeding 10 mg/L during admission still presented with a higher likelihood of death over an average follow-up period of 20 months [45].

Although certain studies have yielded encouraging findings, CRP has not consistently demonstrated itself as a standalone predictor of events. Given the existing treatment approaches for ACS, which include dual antiplatelet, high-intensity statin treatment, and reperfusion, the significance of CRP in regular prognostic estimation for ACS remains uncertain [39].

### 4.2. Interleukin-6 (IL-6)

IL-6, like CRP, has a central role in the inflammatory cascade and has been used as an inflammatory biomarker that may play a significant role in diagnosis, risk stratification, and outcome prediction in patients with AMI. Elevated expression of IL-6 has been observed in induced MI by transcoronary ablation of septal hypertrophy, suggesting potential diagnostic significance [46]. Furthermore, IL-6 shows substantial upregulation in ACS [47] and its levels are linked to adverse cardiac events, highlighting its potential as a therapeutic target in unstable ischemic heart disease [48]. IL-6 receptor antagonists have been found to improve the inflammatory response and the release of cTn after PCI in patients with NSTEMI. This improvement is independent of the inhibition of endothelial cell activation [49].

### 4.3. Myeloperoxidase (MPO)

Myeloperoxidase (MPO) is an enzyme involved in the immune system response and is excreted from neutrophils and macrophages into the extracellular fluid during inflammation [50]. MPO is implicated in the pathophysiology of numerous diseases including atherosclerosis. Is associated with oxidation of LDL cholesterol particles and formation of foam cells; moreover, it has been also studied as a possible therapeutic target against cardiovascular diseases [50,51]. Interestingly, expression of MPO by macrophages is increased at the later stages of atherosclerosis contrary to the initial atherosclerotic lesions (i.e., fatty streaks) in a process controlled by proinflammatory mediators such as granulocyte macrophage colony-stimulating factor (GM-CSF) [52].

Regarding diagnosis of ACS, measurement of MPO at 6 h has displayed a significant diagnostic capability discriminating patients presenting with ACS from patients with other diagnoses of cardiovascular disease [53]. Furthermore, there is an association between MPO levels and the presence of unstable coronary artery plaques as well as the likelihood of future cardiovascular events [54]. Specifically, elevated serum levels of MPO in individuals experiencing ACS have been linked to future cardiovascular events and could identify those at risk for adverse events [55]. Accordingly high levels of MPO on admission could indicate the patients with ACS at risk for complications during their index hospitalization such as HF, arrhythmias and renal failure [56]. Actually, MPO levels have demonstrated an inverse association with left ventricular systolic function in patients hospitalized due to ACS [56]. Moreover, evidence from a meta-analysis of 13 studies has displayed that MPO could predict mortality of patients with ACS and especially for smokers whereas female gender depicted an inverse association with mortality and recurrence of MI; nevertheless, MPO had great prognostic capability irrespectively of other classic cardiovascular risk factors such as age, hypertension and diabetes [57]. Prognostic capability of MPO for future cardiovascular events has been also confirmed by a meta-analysis of 27 studies of patients with ACS [58]. What is more, higher expression of MPO could identify also patients with NSTEMI at risk of major adverse cardiovascular events (MACE) at the first year and particularly for patients > 65 years of age and NT-proBNP levels beyond 1000 pg/mL [59]. Better understanding of the diagnostic and prognostic capability of MPO for patients with ACS could facilitate the development of future clinical studies and possible therapeutic inhibition of the pathways of MPO-related inflammation and oxidative stress [51].

## 5. Biomarkers Associated with Thrombosis-Fibrinolysis

### 5.1. D-Dimers

Whereas the d-dimers have been traditionally established as the diagnostic marker for venous thromboembolism, recently the potential for the diagnosis and prognosis of ACS has been brought to attention due to the coronary artery thrombosis characterizing MI [60]. A recent study considered this biomarker as a potential diagnostic tool for patients with MI and UA; indeed, the determined cut-off to differentiate MI from UA was 548 ng/mL, with 91.2% sensitivity and 63.4% specificity [60]. This cut-off value is compatible with the one from the Bayes-Genis et al. study, where d-dimers > 500 μg/L were diagnostic for MI [61]. In contrast, a recent study found the diagnostic ability of dimers to differentiate between MI and chest pain of non-coronary aetiology to be moderate unless the values exceed the 95th percentile [62]. However, the same study proved the d-dimer’s prognostic ability for MI recurrence (*p* = 0.0333) and all-cause death (*p* < 0.0001) [62]. Finally, a meta-analysis of 5 studies with a mean follow-up of 13.2 months found high d-dimer levels associated with a higher hospital stay, more long-term adverse outcomes, and with the No Reflow Phenomenon (NRP) in STEMI patients after revascularization [63].

### 5.2. Fibrinogen

Fibrinogen (FIB), an early-identified clotting factor, is produced in the liver, and affects processes such as clot formation, platelet aggregation, and the fibrinolysis system. It contributes to the inflammatory response via interactions with cytokines, impacting cardiovascular disease progression. Furthermore, FIB degradation products can induce coronary artery restenosis by promoting smooth muscle cell proliferation [64]. It was discovered that individuals with ACS following PCI who have high baseline fibrinogen levels are at increased risk of MACE within two years [65]. The pathways through which fibrinogen contributes to increased cardiovascular risk can be elucidated as follows: Firstly, fibrinogen stimulates the aggregation of platelets. Moreover, elevated fibrinogen concentrations force the creation of fibrin and elevate plasma viscosity. Lastly, fibrinogen actively engages in inflammatory responses, with its levels rising under inflammatory conditions [66].

## 6. Additional Biomarkers

Beyond the most well-studied biomarkers with clinical applications in the ACS, several other serum biomarkers are continuously evaluated for their diagnostic or prognostic ability in the setting of myocardial infarction (Table 2).

### 6.1. Cystatin C (CysC)

Cystatin C (CysC) is a small 13 kDa molecular peptide and a cysteine protease inhibitor secreted by cells with a nucleus into the bloodstream. It is then streamed freely through the kidney glomerulus and almost thoroughly catabolized into amino acids in the proximal tubule without being secreted. A decline in estimated Glomerular Filtration Rate (eGFR) correlates with a decreased CysC, appointing CysC as a possible biomarker along with creatinine for kidney disease [90]. In fact, CysC interrelation with a worsening renal function as a predictor for mortality has actually been studied in the case of ACS: CysC peaked on the 3rd day after the ACS, whereas creatinine peaked on the 6th day. CysC also was significantly higher than creatinine during the 11.5-month follow-up, therefore predicting mortality or ACS repetition independently of the GRACE score [67]. Correa et al. emphasized a significant association between CysC and long-term risk (2.5 years) of Coronary Vascular Disease (CVD), HF hospitalization or MI for ACS patients [68]. Further, a recent meta-analysis established a significant link between CysC and all-cause death in ACS and MACE but claimed no significant prognostic value for recurrent MI (HR = 1.71 [95%CI: 0.99–2.97]) [69]. This was corroborated in another study, where CysC was prognostic for all-cause death in a 4-year observation interval, but no differences were noted regarding the incidence of non-fatal MI, stroke, UA, and unplanned revascularization [91]. Finally, regarding the prognosis after coronary revascularization in ACS patients, a meta-analysis claimed that higher CysC levels after a PCI were significantly related to mortality and MACE [70], while a different article acknowledged CysC as a possible predictor for the NRP after PCI [92]. Interestingly, high CysC levels before and after Coronary Artery By-Pass Graft Operation (CABG) could significantly relate to renal and cardiovascular effects postoperatively [93], an observation rebutted in the beforementioned meta-analysis with no statistically significant correlation between CysC levels after a CABG with MACE and mortality [70].

### 6.2. Heart-Type Fatty Acid Binding Protein (hFABP)

hFABP, a small soluble molecule normally found in the cardiomyocyte cytoplasm without being cardiac-specific, is responsible for transporting fatty acids [94]. hFABP is released in large concentrations into the plasma after injury, (usual cut-off 5–7 ng/mL), starts rising within 1 h of injury and peaks at 6–8 h (whereas cTn rises after 4–6 h), implicating a possible value for early MI and reinfarction detection. Multiple studies claim that hFABP has a higher sensitivity than single cTn (esp. at 3–6 h after MI), although there is a large heterogeneity between results, probably due to small patient pools, variable cut-off values, different assays, and time of testing [94,95]. In particular, a study has emphasized the ability of hFABP to rule out MI early and characterize up to 40% of patients as low-risk when combined with hs-cTn T [71]. This finding was supported by Van Hise et al.; however, the necessity of an additional biomarker was questioned, as hs-cTn I alone had similar sensitivity and higher specificity than the hFABP and troponin combination [72]. Similarly, in another study, whereas hFABP combined with cTnT at admission improved sensitivity, the same results were reproduced with the serial sampling of troponin at 0 and 90 min, securing the superiority of hs-cTn as a single biomarker for MI [73]. Therefore, the most promising diagnostic ability of hFABP agreed upon in various studies is the possibility of an early MI rule out, which under the results of hs-cTn I is questioned [71,72,74]. Finally, there is discordance between studies regarding the prognosis of mortality by the biomarker, possibly due to different sampling times and populations requiring further research [94,96].

### 6.3. Endocan

Endocan, otherwise endothelial cell-specific molecule 1, is a soluble dermatan sulfate proteoglycan excreted by the activated endothelial cells of vessels. Endocan is upregulated by a variety of proinflammatory cytokines (Tumor Necrosis Factor-a (TNF-α), Interleukin-1β (IL-1β), Vascular Endothelial Growth Factor-a (VEGF-a)), whereas it is implicated in the binding of white blood cells to the endothelium and in inflammatory processes by inducing the levels of Vascular Cell Adhesion Molecule (VCAM), Intercellular Adhesion Molecule (ICAM), and E-selectin [97]. As a result of these mechanisms, endocan has been considered a potential marker for many pathologies, namely sepsis, lung and kidney diseases, preeclampsia, and chronic heart failure [98]. Regarding cardiovascular disease, endocan can potentially act as a surrogate marker for hypertension [99], coronary artery disease (CAD), coronary slow flow [100], angina, and subclinical atherosclerosis [101,102]. Ziaee et al. [75] assessed endocan after a STEMI and UA/NSTEMI and concluded that endocan is independently correlated with MACE, increased thrombolysis in MI (TIMI) risk score and is found in higher levels in STEMI compared to the NSTEMI/UA group. The prognostic role of endocan was also examined in another study, where endocan levels were elevated in the presence of STEMI compared to controls and were associated with increased cardiovascular risk and a high SYNTAX score [76]. Interestingly, due to its pro-inflammatory properties, endocan could possibly assist in predicting the NRP following PCI in STEMI patients, as its levels were significantly increased in the NRP (+) group compared to the control [77]. In contrast, patients undergoing CABG after an ACS had diminished endocan serum levels upon successful reperfusion [78].

### 6.4. Galectin

Galectin-3, a β-galactoside-binding lectin, is predominantly secreted by macrophages and is expressed in a variety of tissues, such as cardiac, renal, hepatic, pulmonary, and vascular tissues. Galectin induces healing and fibrosis by differentiating fibroblasts into myofibroblasts, synthesizing collagen I and III, and contributing to scar formation [103,104]. Galectin-3 is additionally implicated in atherosclerosis as it is upregulated in unstable plaques and attracts monocytes, therefore propagating inflammation [105]. Galectin-3 has been linked to CAD [106] and has been proposed as a prognostic biomarker for HF [104]. A recent meta-analysis examined the interrelation of galectin-3 with MACE following a MI, concluding on: a. a significant negative association between galectin and Left Ventricular Ejection Fraction (LVEF) during and after the MI; b. a non-significant inverted interrelation between galectin and the infarct size; and c. a significant prediction of MACE and all cause-mortality with higher galectin-3 levels [80]. A multitude of studies have emphasized the possible use of galectin as a risk stratification biomarker for MI outcomes. In a study, increased levels sampled 2 days after a MI translated to increased mortality in 5-year follow-up as well as a higher incidence of HF; all associations were found independent of troponin T levels [81]. Similarly, elevated galectin 3 binding protein during a MI correlated with markers of inflammation (IL-1β, fibrinogen, and high-sensitivity CRP), and in a 12-month follow-up, galectin 3 binding protein was associated with increased risk of angina or reinfarction and galectin-3 with all-cause mortality [82]. Another study observed no differences in galectin levels between STEMI and NSTEMI at baseline; however, a positive correlation with hyperlipidemia and carotid atherosclerosis was observed, and during follow-up only patients who did not have a subsequent MI, PCI, CABG, or stroke had a decline in galectin-3 [83]. Finally, galectin couldn’t differentiate between stable angina, NSTEMI, and STEMI in a group of patients; however, in an early stage (1–5 days), it was correlated with atherosclerotic factors (hypertension history and triglycerides), and on the 30-day follow-up, galectin-3 was predictive of diastolic dysfunction and LV remodeling [84].

### 6.5. Soluble Suppression of Tumorigenicity (sST2)

Soluble Suppression of Tumorigenicity 2 factor (sST2), an isoform of ST2, is a portion of the IL-1 receptor family, with its levels increasing in inflammatory diseases as well as in several heart diseases. sST2 is produced by stretching cardiomyocytes and fibroblasts and binds its ligand interleukin-33 (IL-33), not allowing for the desired anti-inflammatory and antifibrotic interaction of IL-33 and the STL2 ligand (STL2L) isoform (transmembrane receptor) [107,108]. ST2 therefore acts as a decoy receptor, and the sST2/IL33 complex may be associated with cardiomyocyte hypertrophy, fibrosis, and remodeling [87,107]. Numerous studies have already proven the strength of sST2 as a prognostic marker for HF, with a proposed cutoff for life-threatening cardiovascular events of 35 ng/mL [107,109]. Aleksova et al., propose specific algorithms both for HF and MI: a. Diagnosis of acute decompensated HF (ADHF) is fairly common (40%) for sST2 > 35ng/mL with values > 70 ng/mL requiring hospitalization; b. Prognosis of Type 1 MI: sST2 can assist with treatment decisions as for <35 ng/mL standard care is proposed, 35–70 ng/mL adverse remodeling is likely and for >70 ng/mL fibrosis is commonly activated and aggressive therapies are needed; and c. Prognosis of Type 2 MI: similar cutoffs and clinical decisions to Type 1 MI [110]. Regarding the ACS, Jenkins et al. suggested that sST2 is associated with age, female sex, and co-morbidities and categorized the sST2 values into 3 tertiles, with patients in the 3rd tertile presenting with a sixfold hazard of mortality and heart failure at 5 years. sST2 in their study did not have a significant relationship with troponin and ECG; therefore, it can be assumed that ST2 as a biomarker expresses different pathophysiologic processes from myocardial injury and ischemia/infarcted burden [85]. Similarly, in a cohort study with 95 STEMI patients enrolled, the mean sST2 was significantly higher in MI patients than in controls whereas for values above the medium, significantly more cardiac adverse effects, and especially acute heart failure, occurred [86]. These findings are, however, challenged in a recent meta-analysis of 16 studies: out of 3 assigned groups (1. ischemic heart disease; 2. MI; and 3. HF), only the HF group had a significant increase in sST2 levels compared to healthy subjects [87]. Finally, high sST2 values have a significant predictive role in the NRP or impaired myocardial reperfusion after a PCI in STEMI patients [111,112].

### 6.6. Micro-RNA

MicroRNAs (miRNAs) are a group of non-coding Ribonucleic Acids (RNAs) that play a significant role in controlling gene expression. Dysregulated miRNA expression is linked to numerous diseases. Interestingly, miRNAs can be released into extracellular fluids, where they can act as potential biomarkers for various conditions and also function as signaling molecules, facilitating cell-to-cell communication [113]. Many research investigations have demonstrated the role of miRNAs in cardiovascular diseases, controlling a wide range of processes such as cardiomyocyte death, cell growth, inflammation, and blood vessel formation [114]. The initial molecules demonstrated to hold prognostic relevance concerning mortality were miRNA-133a and miRNA-208b. These microRNAs were associated with a significant rise in all-cause mortality at 6 months following AMI [115]. Research has demonstrated that miRNA-145 can serve as a prognostic biomarker for cardiovascular mortality and the progression of heart failure [116]. MiRNAs deriving from the cardiac cells, namely miR-1, miR-195, miR-133, miR-126, miR-16, miR-590, miR-199, miR-143, miR-208a, miR-499, miR-27-b, miR-497, miR-126, miR-30-d, miR-208b, miR-15a/b, and miR-16-1/2, play crucial roles in supervising the growth of the cardiac system. Of particular interest are miR-1 and miR-133, which are transcribed together in high amounts specifically in the heart but have contrasting functions in the tissue by promoting cell proliferation while inhibiting cardiac differentiation. Conversely, miR-499 and miR-208, expressed at lower levels in the heart, demonstrate greater specificity for cardiac injury compared to skeletal muscle. miRNAs can enhance genome expression by attaching to promoter sequences and targeted regions; as a result, numerous miRNA transcription compositions are implicated in key pathways related to ACS. These miRNAs are involved in oxidative stress, inflammation, apoptosis, fibrosis, and cardiac remodeling processes that contribute to the pathophysiology of ACS [117]. Serum miR-483-5p, has been recently studied as a potential diagnostic marker for ACS and for its ability to predict major adverse cardiac events after PCI. It was shown that higher levels of miR-483-5p were present in ACS subjects and were linked to the severity of the condition. The miR-483-5p test effectively distinguished ACS patients from healthy controls and differentiated AMI patients from ACS patients. Patients with elevated miR-483-5p had a higher likelihood of experiencing MACE, and miR-483-5p was a strong indicator of MACE incidence after PCI [89].

Measuring plasma miR-22 shortly after admission may serve as a diagnostic tool for MI and a predictor of left ventricular remodeling. However, the reliability of this approach may be influenced by factors like diabetic status and blood parameters, paving the way for further research to enhance patient care and interventions [118].

Due to their ability to control numerous genes through various signaling pathways, miRNAs hold significant promise as innovative therapeutic tools, with miRNA-based approaches extensively applied in areas such as angiogenesis, atherosclerosis, ischemic injury, vascular remodeling, hypertrophy, and fibrosis [119]. MiR-195-3p may play a pivotal role in the development of cardiac fibrosis and dysfunction following a heart attack. Inhibiting miR-195-3p might be a valuable approach for preventing cardiac fibrosis and maintaining heart function after a heart attack [120]. A recent study examined potential gene signatures associated with cardioprotection. The analysis identified 91 differentially expressed genes that may have relevance to AMI. Furthermore, the analysis highlighted the involvement of miR-660 and STAT1, known to impact AMI severity. These genes and miRNA could be pivotal in rescuing cardiomyocytes from severe damage, offering potential insights for the development of therapeutic strategies in AMI management [121].

While miRNAs show promise as potential key players in the early diagnosis of MI, further research is imperative to establish their potency as cardiac biomarkers and their potential implementation in everyday practice [39]. Lastly, long non-coding RNAs (lncRNAs) and circular RNAs (circRNAs) can also shed light on the diagnosis and treatment of cardiovascular diseases [122].

### 6.7. F2 Isoprostanes

F2 isoprostanes are byproducts of arachidonic acid metabolism and are expressed in various cells, including monocytes, during atherosclerotic procedures due to oxidative stress. Research has revealed elevated levels of these compounds in the urine of patients with UA. As potential biomarkers, F2 isoprostanes hold promise in predicting complications in nonfatal MI as well as the progression of HF and mortality [123].

### 6.8. Soluble Oxidized Low-Density Lipoprotein Receptor-1 (sLOX-1)

Soluble oxidized low-density lipoprotein receptor-1 (sLOX-1) was initially identified in 1997 as a scavenger receptor for modified low-density lipoprotein (LDL) found on the endothelium of blood vessels [124].

The research focused on the relationship between sLOX-1 and ACS in individuals with atherosclerotic CVD. ACS patients presented with augmented sLOX-1 levels compared to chronic coronary syndrome patients and healthy controls. Elevated sLOX-1 levels were independently interrelated with a heightened mortality hazard at both 30 days and 1 year. The link between sLOX-1 and cardiovascular mortality was particularly strong. In ACS patients receiving intracoronary imaging and statin therapy, those with coronary plaque regression at 1 year showed a significant reduction in sLOX-1 levels, and sLOX-1 demonstrated good predictive ability for plaque progression. In conclusion, increased plasma sLOX-1 levels during an ACS are associated with mortality in individuals with CVD, and continually high sLOX-1 is associated with coronary plaque progression in those with a history of atherosclerotic CVD [125].

### 6.9. Growth Differentiation Factor 15

Growth Differentiation Factor-15 (GDF-15), formerly recognized as a Non- Steroidal Anti-Inflammatory Drug (NSAID)—activated gene 1 (NAG-1) and macrophage inhibitory cytokine 1 (MIC-1), belongs to the Transforming Growth Factor β (TGF-β) family but stands apart. It is linked to cardiovascular disease and a range of other conditions such as inflammation, oxidative stress, and cellular stress [126]. Elevated GDF-15 levels have been associated with an increased occurrence of cardiovascular events observed in both cases of ST-segment- elevation MI and non-ST-elevation ACS [127]. Moreover, its levels increase with tissue damage and inflammatory conditions. Another study investigated the correlation between GDF-15 concentrations and the likelihood of repeated cardiovascular events in patients who had achieved stabilization after experiencing ACS. It was found that elevated GDF-15 levels were autonomously associated with a heightened probability of recurrent events, suggesting its viability as an indicator for evaluating forthcoming risk [88].

## 7. Conclusions

In conclusion, biomarkers play a pivotal role in diagnosing, stratifying risk, and assessing the prognosis of ACS. In this review article, we systematically discuss the role of several biomarkers, categorizing them based on their mechanisms of action and involved pathways, such as myocardial injury, neurohormonal activation, inflammation, and thrombosis. While this approach provides valuable pathophysiological insights, it’s important to note that the diagnostic and prognostic significance, as well as the clinical utility of most of the investigated biomarkers, is not well established. Accordingly, hs-cTn stands out, revolutionizing ACS diagnosis due to its exceptional sensitivity and negative predictive value underscoring the importance of ongoing research for the establishment and development of biomarkers with added prognostic or diagnostic value in ACS settings.

## Figures and Tables

**Table 1:** Biomarkers used and application in ACS.

Biomarker	Mechanism of Action-Release	Clinical Application & Abilities
hs-Troponin	Regulates cardiac contraction. Released at myocardium necrosis. T & I isoforms cardiac specific	Modification above 99th URL diagnostic for MI hs-cTn has high sensitivity & NPV at cost of specificity. 5x URL increase→high PPV (>90%) for type 1 MI ‘Rule-out’ & ‘rule-in’ MI algorithms Differentiate NSTEMI & UA Rises in 3–12 h, peak at 24 h. Prognosis of all-cause mortality
CK-MB	Released at myocardium necrosis. CK-MB isoenzyme mostly at cardiac muscle (low levels in skeletal muscle)	Diagnosis: Rises after 4–6 h & peaks at 24 h Normal at 48–72 h→detects reinfarction. NPV 97% & sensitivity 91% at first 6 h Prognostic for infarct size, wall motion abnormalities, mortality, HF, possibly LV remodeling, CI-AKI
Cystatin C	Protease inhibitor secreted by nucleated cells. Filtered through glomerulus and catabolized in proximal tubule. Associated with Egfr	Prognostic/risk stratification for all-cause mortality, HF hospitalization, CVD after ACS Peaks at 3rd day after ACS (vs. 6th Creatinine) Prognostic for NRP, MACE & mortality after PCI
H-FABP	Released from cytoplasm after cardiac injury and necrosis	High sensitivity at decreased cutoffs (4 μg/L) Early biomarker (<1 h), reinfarction detection Possible value in early ruling out MI
Endocan	Endothelial dysfunction & activation Inflammation	Risk stratification and Prognosis of MACE, high SYNTAX score Possibly indicates reperfusion after PCI or CABG Possibly different levels in STEMI vs. NSTEMI/UA
Galectin	Cardiac remodeling and fibrosis (fibroblasts→myofibroblasts & collagen synthesis) Plaque Destabilization in CVD	Prognosis of MI and HF Risk stratification (LVEF, MACE, mortality, HF, remodeling) Interrelated with atherosclerosis & inflammation. Possible therapeutic target
sST2	Decoy receptor for sST2/IL-33 interaction cardiac fibrosis, hypertrophy and remodeling	Prognostic factor ADHF (>35 ng/mL) Therapeutic guidance in Type 1 & 2 MI (>35 ng/mL likely adverse remodeling & >70 ng/mL aggressive treatment) Prognostic for ACS (mortality, HF, remodeling) Prognostic for reperfusion & NRP after PCI
D-dimers	Breakdown of fibrin clot by plasmin at the site of coronary artery thrombosis	Possibly diagnostic for MI and differentiate from UA (>500 ng/mL) Prognostic for recurring MI, all-cause mortality, in hospital complications, NRP
CRP	Acute-phase inflammation	Prognostic factor of future myocardial infarction and stroke, levels > 3 mg/L upon discharge: increased risk of readmission within 1 year for recurrent cardiovascular instability or myocardial infarction
micro-RNA	Control of gene expression, oxidative stress, inflammation, apoptosis, fibrosis, and cardiac remodeling processes	Predictive factor for cardiovascular mortality and the development of heart failure
GDF-15	Increases in tissue damage and inflammation	Risk predictor
Fibrinogen	Clot formation, platelet aggregation, fibrinolysis, inflammation	Induce coronary artery restenosis, baseline levels: increased risk of cardiovascular events within 2 years

ACS: Acute Coronary Syndrome; ADHF: Acute Decompensated Heart Failure; CABG: Coronary Artery Bypass Grafting; CI-AKI: Contrast Induced Acute Kidney Injury; CK-MB: Creatine Kinase-MB; CVD: Cardiac Vascular Disease; eGFR: estimated Glomerular Filtration Rate; GDF-15: Growth Differentiation Factor-15; HF: Heart failure; hs-cTn: high sensitivity troponin; LV: Left Ventricle; MACE: Major Adverse Cardiac Events; MI: Myocardial Infarction; NSTEMI: non-ST-elevation Myocardial Infarction; NPV: Negative Predictive Value NRP: No Reflow Phenomenon; PCI: Percutaneous Coronary Intervention; PPV: Positive Predictive Value; UA: Unstable Angina; URL: Upper Reference Limit.

**Table 2:** Studies on potential biomarkers regarding ACS.

Biomarker	Study	Type	Population	Results	Concentrations
Cystatin C (CysC)	Brankovic et al., 2020 [67]	BIOMArCS prospective multicenter study	Case cohort of 844 patients after ACS for 1 year follow-up	CysC independent of GRACE score and associated with mortality, non-fatal MI & revascularization due to angina	CysC at any time associated with endpoint (HR [95% CI]: per 1SD increase of ^2^logCysC: 1.79 [1.21–2.63], *p* = 0.006)
	Correa et al., 2018 [68]	Double- blind clinical trial	4965 random, hospitalized for ACS patients from SOLID-TIMI 52 trial	Strong correlation with Creatinine & eGFR Elevated CysC- 89% higher risk of CVD, HF hospitalization, 44% of MACE, 28% of MI Q4: x5 risk of CVD or HF, >x2 MACE	Quartiles of CysC: Q1 < 0.78, Q2 = 0.78–0.88, Q3 = 0.88–1.03, Q4 > 1.03 mg/mL
	Sun et al., 2021 [69]	Meta-analysis	10 studies	Significant correlation of high level CysC with all-cause mortality & MACE but not significant with recurrent MI	High Q4 and low Q1 quartiles from each study
	Chen et al., 2022 [70]	Meta-analysis	8 studies with 7394 patients after PCI or CABG	↑cystatin significant relation with MACE & mortality after PCI Non-significant after CABG	-
hFABP	Young et al., 2016 [71]	Feasibility study	1079 patients, 248 with MI	hFABP + hs-cTn can identify up to 40% patients as low risk at presentation	hFABP < 4.3 ng/mL + hs-cTn I < 10.0 ng/L + (-) ECG (>99% sensitivity)
	Van Hise et al., 2018 [72]	Cohort study	1230 patients, 112 with MI	h-FABP, hs-cTn and ECG has high accuracy and can rule out more patients	hFABP + hs-cTn T (100% sensitivity + 32.4% specificity) hFABP and hs-cTn I (99.1% sensitivity + 43.4% specificity) hs-cTn I alone higher specificity 68.1%
	Collinson et al., 2013 [73]	Randomized controlled trial	850 patients with chest pain + (-) ECG sampled on admission + 90 min	Hs-cTn best single marker, further info on hFABP required	H-FABP + cTn T/ cTn I (sensitivity 0.78–0.92) at 2.5 μg/L cut-off (single troponin at 2 samples 0.78–0.95)
	Dupuy et al., 2015 [74]	Prospective cohort study	181 patients, 47 with MI (31 NSTEMI) within 12 h	HFABP + hs-cTn T increased sensitivity (+13%) and NPV (+3%) for NSTEMI hFABP lower diagnostic accuracy than hs-cTn T	5.8 ng/mL cutoff (sensitivity of 97% + NPV of 99%)
Endocan	Ziaee et al., 2019 [75]	Cross- sectional and prospective	320 patients with ACS: 160 with STEMI and 160 with UA/NSTEMI	Significant positive correlation between endocan levels and TIMI risk score and MACE.	Optimal cutoff values to predict clinical end points: 3.45 ng/mL in STEMI (80% sensitivity and 72% specificity) and 2.85 ng/mL in NSTEMI/UA (74% sensitivity and 67% specificity)
	Kundi et al., 2017 [76]	Cross- sectional	133 patients: 88 patients with STEMI and 45 patients with normal coronary arteries	Elevated in STEMI and positively correlated with hs-CRP and SYNTAX score	Cutoff value to predict STEMI: 1.7 ng/mL (76.1% sensitivity 73.6% specificity)
	Dogdus et al., 2021 [77]	Cross- sectional	137 STEMI patients undergoing PCI: 45 NRP (+) & 92 NRP (-)	Endocan, initial troponin I, Triglyceride and high-grade thrombus burden were independent predictors of NRP	Cutoff value to predict NRP: >2.7 ng/mL (89.6% sensitivity and 74.2% specificity)
	Cimen et al., 2019 [78]	Cross-sectional	35 ACS patients undergoing CABG	Significant decrease in serum hs-CRP and endocan levels (372.8 ng/mL vs. 320.2) after CABG (*p* < 0.05)	-
	Qiu et al., 2016 [79]	Cross-sectional	216 patients with ACS and 60 controls	Endocan significantly increased in ACS group. STEMI vs NSTEMI: (38.2 [14.4, 78.5] vs 10.5 [2.7, 32.6] ng/mL)	-
Galectin	Tian et al., 2019 [80]	Meta-analysis	2809 patients (10 studies)	Significant negative correlation between galectin & LVEF Non-significant correlation between gal-3 & infarct size Galectin associated with high mortality	-
	Asleh et al., 2019 [81]	Population based cohort study	1342 patients at time of MI	Tertile 2 & 3: 1.3 & 2.4 increased risk of death 1.4 & 2.3 risk of HF	Gal-3 cut-offs in 3 tertiles: 1: <15.1 ng/mL 2: 15.1–22.4 ng/mL 3: >22.4 ng/ml
	Gagno et al., 2019 [82]	Prospective cohort study	469 patients with MI (60% STEMI) with 12 month follow up	Galectin associated with all-cause mortality. Gal-3bp correlated with risk of angina/MI	Median Gal-3bp: 9.1 μg/mL Median Galectin: 9.8 ng/mL
	Święcki et al., 2020 [83]	Controlled pilot study	110 MI patients (66 STEMI & 44 NSTEMI) vs control	Galectin↓ at follow up if endpoint occurrence. Galectin > 9.2 ng/mL at discharge→x9 increase of risk of endpoint occurrence	Galectin cut-off ≥9.2 ng/mL (91% specificity & 50% sensitivity) for MACE at follow-up
	Mitić et al., 2022 [84]	Cohort study	89 patients undergoing PCI	Early galectin correlates with atherosclerosis. Day 30 galectin correlates with diastolic dysfunction and LV remodeling.	-
sST2	Jenkins et al., 2017 [85]	Prospective longitudinal cohort	1401 subjects with MI	Mortality increases at 5 yrs: 11.8%, 25.5% & 52% HF at 5 yrs: 11.4%, 23.6% & 44.8% in respective tertiles	3 tertiles: T1: <37, T2: 37–72.3, T3: <72.3 ng/mL ST2
	Hartopo et al., 2018 [86]	Cohort study	95 STEMI patients & 10 controls	Supramedian sST2 levels in STEMI patients 38.3% versus 12.5% higher incidence of MACE	STEMI vs controls: 152.1 ng/mL vs. 28.5 ng/mL, *p* < 0.01
	Zhang et al., 2020 [87]	Meta-analysis	16 studies	3 groups:1. ischemic heart disease, 2. MI & 3 HF → No statistical significance between control and groups 1 & 2, significant only in 3.	-
D-dimers	Reihani et al., 2018 [60]	Cross- sectional study	75 patients (34 with MI, 41 with UA)	Differentiation of MI (>548) from UA	Cut-off: 548 ng/mL (91.2% sensitivity & 63.4% specificity, *p* < 0.001)
	Koch et al., 2022 [62]	Retrospective study	435 patients with UA, 420 with NSTEMI, 22 NSTEMI, 2680 non coronary cause	PPV for final ACS diagnosis ↑ with d-dimer ↑ Unable to discriminate STEMI from non-coronary cause & UA. ↑d-dimer→↑risk of recurrent MI (esp. Q4) & all-cause mortality	D-dimer concentrations (mg/L): 0.19–0.50 (Q1), 0.51–1.00 (Q2), 1.01–5.00 (Q3), and 5.01–35.00 (Q4).
GDf-15	Bonaca et al., 2010 [88]	Randomized control trial	4162 patients with ACS, follow up for 2 years	significantly higher risk of death and MI	>1362 ng/L, higher rate of death or MI
Fibrinogen	Cetin et al., 2020 [65]	Observational study	261 patients treated with PCI for ACS	FAR predictive of MACE	-
miR-483-5p	Zhao et al., 2023 [89]	Observational study	118 patients with ACS and 75 healthy controls	Serum miR-483-5p levels were higher in ACS patients, high diagnostic value	Cut-off value of 1.292, demonstrated a feasible diagnostic value

ACS: Acute Coronary Syndrome; CABG: Coronary Artery Bypass Grafting; CVD: Cardiac Vascular Disease; eGFR: estimated Glomerular Filtration Rate FAR: fibrinogen-to-albumin ratio; Gal-3bp: Galectin binding protein, GDF-15: Growth Differentiation Factor-15; HF: Heart Failure; LVEF: Left Ventricular Ejection Fraction; MACE: Major adverse cardiac events; MI: Myocardial Infarction; NRP: No-Reflow Phenomenon; MI: Myocardial Infarction; NPV: Negative Predictive Value; PCI: percutaneous coronary intervention; PPV: Positive Predictive Value; TIMI: Thrombolysis in Myocardial Infarction risk score.

## Data Availability

Not applicable.
